# A systematic review and meta-analysis of liver venous deprivation versus portal vein embolization before hepatectomy: future liver volume, postoperative outcomes, and oncological safety

**DOI:** 10.3389/fmed.2023.1334661

**Published:** 2024-01-10

**Authors:** Mohamed Ali Chaouch, Alessandro Mazzotta, Adriano Carneiro da Costa, Mohammad Iqbal Hussain, Amine Gouader, Bassem Krimi, Fabrizio Panaro, Boris Guiu, Olivier Soubrane, Hani Oweira

**Affiliations:** ^1^Department of Visceral and Digestive Surgery, Fattouma Bourguiba Hospital, University of Monastir, Monastir, Tunisia; ^2^Department of Visceral and Digestive Surgery, Institute Mutualist of Montsouris, University of Paris, Paris, France; ^3^Department of General Surgery, Great Western Hospitals NHS Foundation Trust, Swindon, United Kingdom; ^4^Department of Surgery, Perpignan Hospital Center, Perpignan, France; ^5^Department of HPB Surgery and Transplantation, St-Eloi University Hospital, Montpellier, France; ^6^Department of Radiology, St-Eloi University Hospital, Montpellier, France; ^7^Department of Surgery, Universitäts Medizin Mannheim, Heidelberg University, Mannheim, Germany

**Keywords:** liver venous deprivation, portal embolization, liver failure, hepatectomy, surgery, remnant liver volume

## Abstract

**Introduction:**

This systematic review aimed to compare liver venous deprivation (LVD) with portal vein embolization (PVE) in terms of future liver volume, postoperative outcomes, and oncological safety before major hepatectomy.

**Methods:**

We conducted this systematic review and meta-analysis following the PRISMA guidelines 2020 and AMSTAR 2 guidelines. Comparative articles published before November 2022 were retained.

**Results:**

The literature search identified nine eligible comparative studies. They included 557 patients, 207 in the LVD group and 350 in the PVE group. This systematic review and meta-analysis concluded that LVD was associated with higher future liver remnant (FLR) volume after embolization, percentage of FLR hypertrophy, lower failure of resection due to low FLR, faster kinetic growth, higher day 5 prothrombin time, and higher 3 years’ disease-free survival. This study did not find any difference between the LVD and PVE groups in terms of complications related to embolization, FLR percentage of hypertrophy after embolization, failure of resection, 3-month mortality, overall morbidity, major complications, operative time, blood loss, bile leak, ascites, post hepatectomy liver failure, day 5 bilirubin level, hospital stay, and three years’ overall survival.

**Conclusion:**

LVD is as feasible and safe as PVE with encouraging results making some selected patients more suitable for surgery, even with a small FLR.

**Systematic review registration:**

The review protocol was registered in PROSPERO before conducting the study (CRD42021287628).

## Introduction

One of the major issues after extended hepatectomy is the insufficient remnant liver volume and liver function. Since its first report in 1986 (1), portal vein embolization (PVE) has been used to enhance future liver remnant volume (FLR) and prevent post-hepatectomy liver failure (PHLF). The principle is to lead to ipsilateral hepatic atrophy and contralateral future liver hypertrophy. Currently, PVE is considered the standard preoperative manipulation to improve the volume of an adequate FLR and reduce the risk of PHLF (2). However, several studies have shown that liver remnant inadequacy also depends on the future liver parenchymal condition (3). PVE allows up to 70–80% of patients to subsequently undergo major hepatectomy (4, 5). However, the major concern regarding PVE is the dropout of up to 36% of patients because of insufficient FLR hypertrophy or tumor progression within 4-to 6-week intervals between PVE and definitive resection (6, 7). In recent years, alternative strategies have been proposed, such as two-stage hepatectomy with portal vein (PV) ligation, additional ligation of the ipsilateral hepatic artery, and associating liver partition and PV ligation for staged hepatectomy (ALPPS) (8). Two-stage hepatectomy with PV ligation induced a similar FLR to PVE, the ipsilateral artery ligation quickly abounded due to a high rate of liver abscess, and the ALPPS was associated with higher postoperative morbidity as well as inferior long-term survival (9). For these reasons, liver venous deprivation (LVD), and a simultaneous embolization of the portal and hepatic ipsilateral veins have been suggested. Different studies comparing LVD and PVE concluded with controversial results. Considering all these findings, we conducted a systematic review and meta-analysis.

This systematic review and meta-analysis aimed to compare LVD and PVE in terms of FLR, postoperative outcomes, and oncological safety.

## Methods

We conducted this systematic review and meta-analysis according to the PRISMA 2020 (Preferred Reporting Items for Systematic Review and Meta-analysis) (10) and AMSTAR 2 (assessing the methodological quality of systematic reviews) (11) guidelines. The review protocol was registered in PROSPERO before conducting the study (CRD42021287628).

### Electronics searches

The electronic search was conducted on November 30, 2022, with no language restrictions, in the following databases “Cochrane Library,” “PubMed/MEDLINE,” “Excerpta Medica Database,” “Embase,” and “Google Scholar.” Keywords used were: “liver venous deprivation”; “portal vein embolization”; “major hepatectomy”; “future liver remnant”; “liver failure”; “hepatectomy”; “liver failure”; “bilirubin”; “prothrombin”; “surgery”; “outcome,” “morbidity,” and “mortality.” We used the Boolean markers “and” and “or.” The reference lists of the articles obtained were checked for eligible clinical trials.

*Study selection*: Clinical controlled trials (CCT) comparing LVD with PVE before major liver resection were retained.

*Participants/population*: Adults operated on for liver disease and candidates for an induction technique of liver regeneration before major hepatectomy.

*Intervention group*: Single-stage LVD.

*Control group*: PVE before surgery.

*Outcomes*: The different outcomes assessed were the remnant future liver volume (complications of embolization, future liver remnant volume and percentage before embolization, future liver remnant volume and percentage after embolization, future liver remnant volume ratio after embolization, future liver remnant hypertrophy, kinetic growth rate, and failure of resection), postoperative outcomes (3-month postoperative mortality, overall morbidity, Clavien-Dindo complications≥ grade III, blood loss, bile leak, ascites, operative time, post hepatectomy liver failure [defined according to ISGLS (International Study Group of Liver Surgery) or “50–50” criteria (12, 13)], day 5 bilirubin level, day 5 prothrombin time, and hospital stay), and oncological outcomes [three years overall survival (OS) and disease-free survival (DFS)].

*Assessment of study quality and risk of bias*: We used the MINORS (Methodological Index for Non-randomized studies) (14) and the Newcastle-Ottawa Scale (NOS) (15).

*Study selection and data extraction*: Two authors selected studies and extracted data. Disparities were resolved by the senior author.

*Certainly assessment of evidence*: We used the GRADE guidelines to rate evidence quality (16). We considered the study limitations in terms of the constancy of effect, imprecision, indirectness, and publication bias. We assessed the certainty of the evidence as high, moderate, low, or very low. If appropriate, we considered the following criteria for upgrading the certainty of evidence: large effect, dose–response gradient, and plausible confounding effect. GRADEpro GDT software was used to prepare the “Summary of findings tables.” We explained the reasons for downgrading or upgrading the included studies using footnotes and comments.

*Assessment of heterogeneity*: We used the Cochrane Chi^2^ test (Q-test), Tau^2^, of true effects (17) and graphical exploration with funnel plots (18).

*Evaluation of effect size*: We used the Review Manager 5.3.5 statistical package (19). We selected the standardized mean difference (SMD) as an effective measure of continuous data and odds ratios (OR) with 95% confidence intervals (95% CI) for dichotomous data. A random effects model was used. The threshold for significance was set at *p* < 0.05.

## Results

### Literature research

**Figure 1:**
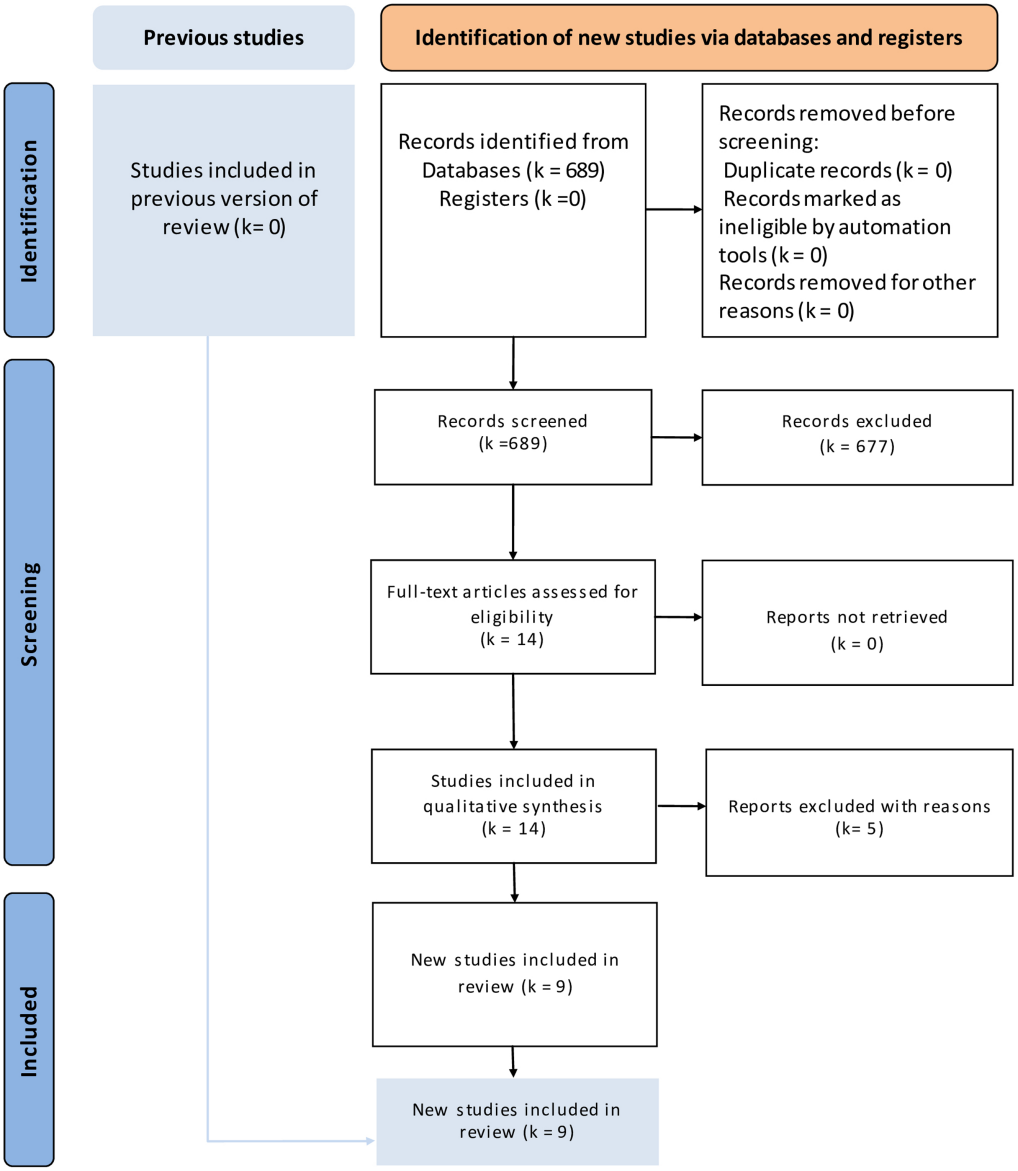
PRISMA flow diagram of the literature research.

The literature search identified fourteen eligible articles (Figure 1) (20–28). Five articles were excluded for the following reasons: two assessed only liver hypertrophy and pathological changes without post-major hepatectomy outcomes (29, 30), one letter to the editor (31), one non-comparative study (32), and one study protocol (33). Finally only nine studies were included in our study (Table 1). These articles were published between 2018 and 2022. They included 557 patients, 207 in the LVD group and 350 in the PVE group. The demographic data are reported in Table 2. The sex ratio was 1.48. The mean BMI ranged from 23.4 kg/m^2^ to 26.3 kg/m^2^ in the LVD group and from 23.8 kg/m^2^ to 25.5 kg/m^2^ in the PVE group. Liver metastases of colorectal cancer were the most frequent indication. Extended right hepatectomy was the most frequent procedure. The delay for volumetric analysis ranged from 17 to 30.5 days and for resection ranged from 32 to 44 days and the follow-up ranged from one to 36 months.

**Table 1:** List of the retained studies.

First author	Journal	Country	Study period	Year of publication	Study design	Number of patients	MINORS	NOS
Böning et al. (20)	Cardiovasc Intervent Radiol	Germany	June 2018 – August 2019	2022	Prospective non-randomized trial / matched pair analysis	28	22	8
Cassese et al. (34)	Journal of Gastrointestinal Surgery	France	May 2015 – December 2019	2022	Retrospective analysis of consecutive patients	33	20	9
Guiu et al. (22)	HepatoBiliary Surgery and Nutrition	France	January 2017 – March 2019	2020	Retrospective study	51	18	7
Heil et al. (23)	British Journal of Surgery	Germany/Belgium/ Spain/Canada/Netherlands/Switzerland	January 2016–December 2019	2021	Prospective non-randomized trial	199	20	8
Hocquelet et al. (24)	Cardiovasc Intervent Radiol	Switzerland/France	204–2018	2018	Retrospective study	12	16	9
Kobayashi et al. (25)	Surgery	Switzerland	2010–2016	2020	Prospective non-randomized trial	60	20	8
Laurent et al. (26)	Annals of Surgery	France	January 2016 – December 2018	2020	Retrospective study	73	18	9
Le Roy et al. (27)	HPB	France	January 2010 – December 2017	2019	Prospective non-randomized trial	72	20	7
Panaro et al. (28)	HepatoBiliary Surgery and Nutrition	France	April 2015 – December 2017	2019	Retrospective study	29	18	6

MINORS, Methodological index for non-randomized studies; NOS, Newcastle-Ottawa Scale.

**Table 2:** Demographic data of the retained studies.

Authors	Number of patients	LVD	PVE	Gender		Age (years)		BMI (kg/m^2^)		Indications				Type of surgery				Time to volumetric analysis (days) (LVD/PVE)	Time to resection (days) (LVD/PVE)	Follow-up (months) (LVD/PVE)
				Male	Female	LVD	PVE	LVD	PVE	Cholangiocarcinoma	HCC	CRCM	Others	RH	ERH	LH	ELH			
Böning et al. (20)	28	14	14	10	18	68.1	65.1	24.1	26.1	-	-	20	8	0	28	-	-	30.5	-	12
Cassese et al. (34)	33	17	16	20	13	58.9	65.2	-		-	-	25		-	-	-	-	-	39	36
Guiu et al. (22)	51	29	22	37	14	62	66	26.3	25.1	7	3	29	2	23	26	-	-	21	32/36	3
Heil et al. (23)	199	39	160	120	79	63	67	24.4	25.2	58	15	104	24	60	79	2	12	17/24	37/41	12
Hocquelet et al. (24)	12	6	6	-		60	62	-		12	-	-	-	0	12	-	-	23,5	-	36
Kobayashi et al. (25)	60	21	39	31	19	65	65	23.4	23.8	10	4	36	-	28	22	-	-	22/26	35	20.3/18
Laurent et al. (26)	73	37	36	51	22	64.4	60.9	25.4	25.5	14	8	43	5	29	25	-	-	28	36/44	1
Le Roy et al. (27)	72	31	41	44	28	66	63	24	24	6	5	18	1	8	15	-	-	26/27	-	3
Panaro et al. (28)	29	13	16	-		-		-		-	11	15	2	0	29	-	-	-	38/37	3

HCC, hepatocellular carcinoma; CCRM, colorectal cancer metastases; RH, right hepatectomy; ERH, extended right hepatectomy; LH, left hepatectomy; ELH, extended left hepatectomy.

### Post embolization results

#### Complications of the embolization

All retained studies reported complication rates after hepatectomy (20–28). This outcome was reported in 16 of 207 patients in the LVD group and 28 of 350 patients in the PVE group. There was no difference between the two groups (OR = 1.44; 95%CI [0.68, 3.03], *p* = 0.34).

#### FLR volume before embolization (mL)

Six studies reported the FLR volume before embolization (22–27). This outcome was reported in 163 patients in the LVD group and 264 patients in the PVE group. There was no difference between the two groups (SMD = 0.02; 95%CI [−0.47,0.52], *p* = 0.92).

#### FLR percentage before embolization (%)

Six studies reported the FLR percentage before embolization (22–27). This outcome was reported in 149 patients in the LVD group and 279 patients in the PVE group. There was no difference between the two groups (SMD = −0.50; 95%CI [−1.10, 0.09], *p* = 0.10).

#### FLR volume after embolization

Five studies reported the FLR volume after embolization (23–27). This outcome was reported in 134 patients in the LVD group and 282 patients in the PVE group. There was a higher FLR volume after embolization in the LVD group (SMD = 0.62; 95%CI [0.06, 1.19], *p* = 0.03).

#### FLR percentage after embolization

Five studies reported FLR percentage after embolization (21, 23–26). This outcome was reported in 120 and 257 patients in the LVD and PVE groups, respectively. There was no difference between the two groups (SMD = 0.53; 95% CI [−0.14, 1.21], *p* = 0.12).

#### FLR remnant liver hypertrophy (%)

Eight studies reported FLR remnant liver hypertrophy after embolization (20–28). This outcome was reported in 194 patients in the LVD group and 334 patients in the PVE group. The FLR volume after embolization was higher in the LVD group (SMD = 0.80; 95%CI [0.39, 1.21], *p* = 0.0001).

#### Kinetic growth

Four studies reported kinetic growth after embolization (21, 23, 25, 27). This outcome was reported in 108 patients in the LVD group and 256 patients in the PVE group. The volume of kinetic growth after embolization was higher in the LVD group (SMD = 2.08; 95%CI [0.61, 3.54], *p* = 0.005).

#### Failure of resection

All the retained articles reported the rate of resection failure (20–28). In the LVD group, twenty-five out of 190 patients failed to undergo resection. In the PVE group, eighty-three patients out of the 334 patients failed to undergo resection. There was no difference between the two groups (OR = 0.59; 95%CI [0.33, 1.06], *p* = 0.08).

#### Failure of resection due to inadequate FLR

All retained articles reported the rate of resection failure due to inadequate FLR (20–28). Three of 207 patients in the LVD group and 23 of 346 patients in the PVE group. We found a lower rate of resection failure due to low FLR in the LVD group (OR = 0.30; 95%CI [0.09, 0.96], *p* = 0.04).

### Postoperative results

#### 3-month mortality

All retained articles reported the 3-month mortality (20–28). It was found in seven of 182 patients in the LVD group and 29 of 268 patients in the PVE group. There was no difference between the two groups (OR = 0.45; 95%CI [0.18, 1.13], *p* = 0.09).

#### Overall morbidity

Six articles reported the overall morbidity (21, 23, 25–28). It was reported in 72 of 142 patients in the LVD group and 126 of 234 patients in the PVE group. There was no difference between the two groups (OR = 1.07; 95%CI [0.59, 1.97], *p* = 0.82).

#### Clavien-Dindo ≥ grade III

Seven articles reported postoperative major complications (21–23, 25–28). It was reported in seven of 32 of 169 patients in the LVD group and 71 of 126 patients in the PVE group. There was no difference between the two groups (OR = 0.71; 95%CI [0.43, 1.17], *p* = 0.18).

#### Operative time

Five studies reported the operative time (21, 23, 25, 27, 28). This outcome was reported in 110 patients in the LVD group and 201 patients in the PVE group. There was no difference between the two groups (SMD = 0.02; 95%CI [−0.41, 0.46], *p* = 0.91).

#### Blood loss

Six studies reported blood loss during hepatectomy (21, 23, 25–28). This outcome was reported in 142 patients in the LVD group and 234 patients in the PVE group. There was no difference between the two groups (SMD = 0.18; 95%CI [−0.04, 0.39], *p* = 0.11).

#### Bile leak

Three studies reported the bile leak rates (21, 26, 28). It was reported in five of 62 patients in the LVD group and six in 63 patients in the PVE group. There was no difference between the two groups (OR = 0.80; 95%CI [0.18, 3.53], *p* = 0.77).

#### Ascites

Two articles reported on postoperative ascites (21, 26). It was reported in six of 49 patients in the LVD group and five of 48 patients in the PVE group. There was no difference between the two groups (OR = 1.19; 95%CI [0.33, 4.32], *p* = 0.80).

#### Post hepatectomy liver failure

The rate of PHLF has been reported in eight studies (20–24, 26–28). Twenty-one of 162 patients in the LVD group presented with PHLF versus 50 of 238 patients in the PVE group. There was no difference between the two groups (OR = 0.77; 95%CI [0.34, 1.79], *p* = 0.55).

#### Day 5 bilirubin level

Four studies investigated day 5 bilirubin levels (22–24, 26). They compared 98 and 165 patients in the PVE and HVD groups, respectively. There was no difference between the two groups (SMD = 3.06; 95%CI [−4.08, 10.20], *p* = 0.40).

#### Day 5 prothrombin

Three studies reported day 5 prothrombin values (22, 24, 26). They compared 63 and 56 patients in the PVE and LVD groups, respectively. Prothrombin rates were higher in the LVD group (SMD = 6.85; 95%CI [0.42, 13.28], *p* = 0.04).

#### Hospital stay

Five studies reported hospital stay (23–27). They compared 116 and 208 patients in the PVE and LVD groups, respectively. There was no difference between the two groups (SMD = −5.31; 95%CI [−12.17, 1.55], *p* = 0.13).

### Oncological outcomes

#### Three years’ OS

Two studies reported the three years’ OS (21, 25). It was reported in 25 of 37 patients in the LVD group and 35 of 46 patients in the PVE group. There was a similar three-year OS (OR = 0.70; 95%CI [0.22, 2.20], *p* = 0.54).

#### Three-year DFS

Two studies reported the three-year DFS (21, 25). It was reported in 14 of 37 patients in the LVD group and four of 46 patients in the PVE group. There was a higher three-year DFS in the LVD group (OR = 5.88; 95%CI [1.05, 32.87], *p* = 0.04).

### Quality assessment of the included studies and reporting of the effects of LVD

Table 3 summarizes the different findings of the pooled analysis. The MINORS and NOS scores were reported in Table 1. A Summary of the evidence was presented in Tables 4–6. This review showed that when LVD is compared with PVE:

- The post-embolization results (Table 4), it may ensure a higher FLR volume after embolization, a percentage of FLR hypertrophy, lower failure of resection due to low FLR, and faster kinetic growth. We do not know if it leads to additional complications related to the embolization, FLR percentage of hypertrophy after embolization, or failure of resection because the evidence was very uncertain.
- For the postoperative outcomes (Table 5), it may ensure a higher day 5 prothrombin time. We do not know if it leads to additional 3-month mortality, overall morbidity, major complications, operative time, blood loss, bile leak, ascites, post hepatectomy liver failure, day 5 bilirubin level, and hospital stay because the evidence was very uncertain.
- For the oncological outcomes (Table 6), it may ensure a higher 3 years of DFS. We do not know if it leads to higher three-year OS because the evidence was very uncertain.

**Table 3:** Different outcomes of the pooled analysis.

Outcomes	Number of studies	Number of patients	LVD	PVE	OR/SMD	95% IC	Tau^2^	*p*
Post embolization results								
Complications of the embolization	9	44/557	16/207	28/350	1.44	0.68, 3.03	0	0.34
FLR volume before embolization (mL)	6	427	163	264	0.02	−0.47, 0.52	0.29*	0.92
FLR percentage before embolization (%)	6	428	149	279	−0.50	−1.10, 0.09	0.45*	0.10
FLR volume after embolization (mL)	5	416	134	282	0.62	0.06, 1.19	0.29*	0.03
FLR percentage after embolization (%)	5	377	120	257	0.53	−0.14, 1.21	0.48*	0.12
FLR hypertrophy (%)	8	528	194	334	0.80	0.39, 1.21	0.24*	0.0001
e kinetic growth (%/week)	4	364	108	256	2.08	0.61, 3.54	2.11*	0.005
Failure of resection	9	108/524	25/190	83/334	0.59	0.33, 1.06	0.05*	0.08
Failure of resection due to low FLR	9	26/553	3/207	23/346	0.30	0.09, 0.96	0	0.04
Postoperative outcomes								
3-month mortality	9	36/450	7/182	29/268	0.45	0.18, 1.13	0	0.09
Overall morbidity	6	198/376	72/142	126/234	1.07	0.59, 1.97	0.24*	0.82
Dindo-Clavien ≥ grade III	7	103/422	32/169	71/253	0.71	0.43, 1.17	0	0.18
Operative time	5	311	110	201	0.22	−0.41, 0.46	0.16*	0.91
Blood loss	6	376	142	234	0.18	−0.04, 0.39	0	0.11
Bile leak	3	11/125	5/62	6/63	0.8	0.18, 3.53	0.2*	0.77
Ascites	2	11/97	6/49	5/48	1.19	0.33, 4.32	0	0.80
Post hepatectomy liver failure	8	71/400	21/162	50/238	0.77	0.34, 1.79	0.33*	0.55
Day 5 bilirubin level	4	263	98	165	3.06	−4.08, 10.20	38.08***	0.40
Day 5 prothrombin time	3	119	63	56	6.85	0.42, 13.28	22.60**	0.04
Hospital stay	5	324	116	208	−5.31	−12.17, 1.55	53.31***	0.13
Oncological outcomes								
Three years OS	2	60/83	25/37	35/46	0.70	0.22, 2.20	0.16*	0.54
Three years DFS	2	18/83	14/37	4/46	5.88	1.05, 32.87	0.55*	0.04

LVD, liver venous deprivation; PVE, portal vein embolization; OR, odd’s ratio; SMD, standardized mean difference; IC, interval of confidence; OS, overall survival; DFS, disease free survival. ^*^Low heterogeneity among the studies. ^**^Moderate heterogeneity among the studies. ^***^High heterogeneity among the studies.

**Table 4:** Summary of findings table of the post embolization outcomes.

Outcomes	No. of participants (studies) Follow-up	Certainty of the evidence (GRADE)	Relative effect (95% CI)	Anticipated absolute effects	
				Risk with PVE	Risk difference with LVD
Complications of embolization	557 (9 observational studies)	⨁⨁◯◯ Low	OR 1.44 (0.68 to 3.03)	80 per 1,000	31 more per 1,000 (24 fewer to 129 more)
FLR before embolization (mL)	467 (6 observational studies)	⨁⨁◯◯ Low	-	-	SMD 0.02 higher (0.47 lower to 0.52 higher)
FLR before embolization (%)	428 (6 observational studies)	⨁⨁◯◯ Low	-	-	SMD 0.5 lower (1.1 lower to 0.09 higher)
FLR after embolization (mL)	416 (5 observational studies)	⨁◯◯◯ Very low^a,b^	-	-	SMD 0.62 higher (0.06 higher to 1.19 higher)
FLR after embolization (%)	377 (5 observational studies)	⨁⨁⨁◯ Moderate	-	-	SMD 0.53 higher (0.14 lower to 1.21 higher)
FLR hypertrophy (%)	528 (8 observational studies)	⨁⨁◯◯ Low^a,b^	-	-	SMD 0.8 higher (0.39 higher to 1.21 higher)
e kinetic growth rate	364 (4 observational studies)	⨁⨁◯◯ Low^a,b^	-	-	SMD 2.08 higher (0.61 higher to 3.54 higher)
Failure of resection	557 (9 observational studies)	⨁⨁◯◯ Low	OR 0.59 (0.33 to 1.06)	237 per 1,000	82 fewer per 1,000 (144 fewer to 11 more)
Failed of resection due to low FLR	553 (9 observational studies)	⨁⨁⨁◯ Moderate^a^	OR 0.30 (0.09 to 0.96)	66 per 1,000	46 fewer per 1,000 (60 fewer to 2 fewer)

^*^The risk in the intervention group (and its 95% confidence interval) is based on the assumed risk in the comparison group and the relative effect of the intervention (and its 95% CI). CI, confidence interval; MD, mean difference; OR, odds ratio; SMD, standardized mean difference. GRADE Working Group grades of evidence: High certainty: we are very confident that the true effect lies close to that of the estimate of the effect. Moderate certainty: we are moderately confident in the effect estimate: the true effect is likely to be close to the estimate of the effect, but there is a possibility that it is substantially different. Low certainty: our confidence in the effect estimate is limited: the true effect may be substantially different from the estimate of the effect. Very low certainty: we have very little confidence in the effect estimate: the true effect is likely to be substantially different from the estimate of effect. ^a^Small sample size of patients. ^b^Existing heterogeneity among the different retained studies.

**Table 5:** Summary of findings table of the postoperative outcomes.

Outcomes	No. of participants (studies) Follow-up	Certainty of the evidence (GRADE)	Relative effect (95% CI)	Anticipated absolute effects	
				Risk with PVE	Risk difference with LVD
3-month mortality	450 (9 observational studies)	⨁⨁◯◯ Low	OR 0.45 (0.18 to 1.13)	Low	
				0 per 1,000	0 fewer per 1,000 (0 fewer to 0 fewer)
Morbidity	376 (6 observational studies)	⨁⨁◯◯ Low	OR 1.07 (0.59 to 1.97)	538 per 1,000	17 more per 1,000 (131 fewer to 158 more)
Clavien-Dindo ≥ IIIA	422 (7 observational studies)	⨁⨁◯◯ Low	OR 0.71 (0.43 to 1.17)	281 per 1,000	64 fewer per 1,000 (137 fewer to 33 more)
Operative time	311 (5 observational studies)	⨁◯◯◯ Very low^a,b^	-	-	SMD 0.02 higher (0.41 lower to 0.46 higher)
Blood loss	376 (6 observational studies)	⨁◯◯◯ Very low^a^	-	-	SMD 0.18 higher (0.04 lower to 0.39 higher)
Bile leak	125 (3 observational studies)	⨁◯◯◯ Very low^a,b^	OR 0.80 (0.18 to 3.53)	95 per 1,000	18 fewer per 1,000 (77 fewer to 176 more)
Ascites	97 (2 observational studies)	⨁◯◯◯ Very low^a,b^	OR 1.19 (0.33 to 4.32)	104 per 1,000	17 more per 1,000 (67 fewer to 230 more)
Hepatic failure	400 (8 observational studies)	⨁⨁◯◯ Low	OR 0.77 (0.34 to 1.79)	210 per 1,000	40 fewer per 1,000 (127 fewer to 112 more)
Day 5 bilirubin	263 (4 observational studies)	⨁◯◯◯ Very low^a,b^	-	-	MD 3.06 higher (4.08 lower to 10.2 higher)
Day 5 prothrombin	119 (3 observational studies)	⨁◯◯◯ Very low^a,b^	-	-	MD 6.85 higher (0.42 higher to 13.28 higher)
Hospital stay	324 (5 observational studies)	⨁⨁◯◯ Low	-	-	MD 5.31 lower (12.17 lower to 1.55 higher)

^*^The risk in the intervention group (and its 95% confidence interval) is based on the assumed risk in the comparison group and the relative effect of the intervention (and its 95% CI). CI, confidence interval; MD, mean difference; OR, odds ratio; SMD, standardized mean difference. GRADE Working Group grades of evidence: High certainty: we are very confident that the true effect lies close to that of the estimate of the effect. Moderate certainty: we are moderately confident in the effect estimate: the true effect is likely to be close to the estimate of the effect, but there is a possibility that it is substantially different. Low certainty: our confidence in the effect estimate is limited: the true effect may be substantially different from the estimate of the effect. Very low certainty: we have very little confidence in the effect estimate: the true effect is likely to be substantially different from the estimate of effect. ^a^Small sample size of patients. ^b^Existing heterogeneity among the different retained studies.

**Table 6:** Summary of findings table of the oncological outcomes.

Outcomes	No. of participants (studies) Follow-up	Certainty of the evidence (GRADE)	Relative effect (95% CI)	Anticipated absolute effects	
				Risk with PVE	Risk difference with LVD
Three years OS	83 (2 observational studies)	⨁◯◯◯ Very low^a,b^	OR 0.70 (0.22 to 2.20)	761 per 1,000	71 fewer per 1,000 (349 fewer to 114 more)
Three-year DFS	83 (2 observational studies)	⨁◯◯◯ Very low^a,b^	OR 5.88 (1.05 to 32.87)	87 per 1,000	272 more per 1,000 (4 more to 671 more)

^*^The risk in the intervention group (and its 95% confidence interval) is based on the assumed risk in the comparison group and the relative effect of the intervention (and its 95% CI). CI, confidence interval; MD, mean difference; OR, odds ratio; SMD, standardized mean difference. GRADE Working Group grades of evidence: High certainty: we are very confident that the true effect lies close to that of the estimate of the effect. Moderate certainty: we are moderately confident in the effect estimate: the true effect is likely to be close to the estimate of the effect, but there is a possibility that it is substantially different. Low certainty: our confidence in the effect estimate is limited: the true effect may be substantially different from the estimate of the effect. Very low certainty: we have very little confidence in the effect estimate: the true effect is likely to be substantially different from the estimate of effect. ^a^Small sample size of patients. ^b^Existing heterogeneity among the different retained studies.

## Discussion

This study concluded that the LVD group was associated with higher FLR volume after embolization, percentage of FLR hypertrophy, lower failure of resection due to low FLR, faster kinetic growth, higher day 5 prothrombin time, and higher 3 years DFS. This study did not find any difference between the LVD and PVE groups in terms of complications related to embolization, FLR percentage of hypertrophy after embolization, failure of resection, 3-month mortality, overall morbidity, major complications, operative time, blood loss, bile leak, ascites, PHLF, day 5 bilirubin level, hospital stay, and three years’ OS.

Mortality and morbidity remain major concerns after an extended hepatectomy. This could be related to technical factors causing bile leakage and abscess, or PHLF causing hypoalbuminemia, cholestasis, and synthetic dysfunction (35). Hwang et al. (36), first, proposed sequential PV and ipsilateral hepatic vein embolization in 42 patients. They found a volume increase of 13.3% after PVE versus 28.9% after sequential PVE followed by hepatic vein embolization. In 2016, Guiu et al. (37) concluded that trans-hepatic LVD is feasible, well tolerated, and provides faster and more important hypertrophy of the FLR. In our study, this safety was confirmed by the similar success and complication rates. Furthermore, most complications after LVD were managed conservatively. Only one of the 16 patients died due to infected tumor necrosis. We concluded that the adjunction of ipsilateral embolization of the liver vein to the PV did not lead to additional mortality or morbidity. Our study demonstrated also that after LVD a small number of patients failed to undergo liver resection. However, when we assessed only resection failure due to an inadequate FLR, we found a lower rate in the LVD group: three out of 25 patients (12%) versus 23 out of 82 patients (28%). These findings were confirmed by reporting a higher FLR hypertrophy percentage and FLR volume after embolization. Even more, heterogeneity among the studies could be explained by including left and right hepatectomies, additional embolization of the segment-4 PV, or middle hepatic vein embolization. In addition, no strict volumetric criteria exist for inclusion like mixed tumor types, and cirrhotic and non-cirrhotic patients. Furthermore, pooled data on multiple factors that could affect FLR growth, such as age, malnutrition, obesity, chronic renal failure, and preoperative chemotherapy were not precise (34). To assess the accuracy of FLR hypertrophy, we have compared FLR volume and percentage data before embolization. Another advantage of LVD was faster kinetic growth (2.08%/week). According to Shindoh et al. (38), the kinetic growth rate is a better predictor of postoperative morbidity and mortality than the standardized future liver remnant and degree of hypertrophy. In a subgroup analysis, a kinetic growth rate < 2%/week was associated with higher major complications, PHLF, and 3-month mortality.

In addition, we found that postoperative outcomes directly depend on the FLR size (39). In the case of a small remnant liver, we have a “small for size” liver syndrome (40), causing an increase in portal pressure and blood flow to the remnant liver and mesentery. Consequently, it causes sinusoidal endothelial and Kupffer cell injury and inflammatory cytokine release. Some authors raised the risk of increased blood loss due to the development of “pseudo-Budd-Chiari syndrome” after LVD (24–26). In our study, we did not find a difference in terms of PHLF and ascites. It seems that the surgical procedure was not more challenging after LVD with similar 30-day mortality, morbidity, major complications, blood loss, and operative time. Furthermore, in studies reporting the Pringle manoeuvre, there was no difference between the LVD and PVE groups. This evidence supported the absence of a “pseudo-Budd-Chari syndrome” after hepatectomy.

Regarding oncological data, our study concluded to similar three-year OS with a higher 3-year DFS. Two other studies, with longer follow-ups, of Azoulay et al. (40) and Elias et al. (41) did not find a difference in 5-year OS. Then, we concluded that LVD ensures at least similar oncological safety.

On the other hand, we should consider a brief comparison between LVD and ALPPS that present an additional option for these area. LVD and ALPPS differ in their approach, speed of liver regeneration, and risk profiles (8). The choice between these techniques depends on various factors including the patient’s condition, extent of liver disease, and the surgeon’s expertise. LVD aims to accelerate liver hypertrophy by blocking the blood supply to the diseased part of the liver, thereby redirecting blood to the healthier part. However, ALPPS seeks to rapidly induce hypertrophy of the future liver remnant by surgically dividing the liver and ligating the blood supply to the diseased portion. Generally, LVD has a slower hypertrophy response compared to ALPPS and ALPPS Known for inducing rapid liver hypertrophy, often within a week or two (9). On the other side, we should consider that LVD potentially reduces the risk of postoperative complications compared to ALPPS, but this can vary based on patient-specific factors and ALPPS is associated with higher morbidity and mortality rates due to the invasiveness of the procedure. For these reasons, LVD is often used in patients where traditional PVE is not effective or in cases with extensive liver disease and ALPPS is typically considered for patients with extensive liver tumors or small future liver remnants where traditional approaches like PVE might not induce sufficient hypertrophy.

Our results should be interpreted within the context of these limitations. Owing to the absence of RCTs, we only included CCTs and our conclusions should be considered with cautions. Comparison of liver volumetry and function should be performed using reproducible and comparable methods, and large amounts of data were required to obtain a definite opinion. One of the missing outcomes to evaluate was the postoperative remnant liver function. Only the comparative study by Guiu et al. (22) assessed FLR function using scintigraphy. In our study, referring to the “50–50” criteria, we have found higher prothrombin time on day 5 post hepatectomy in the LVD group which confirms to some degree these findings of retrospective studies (22, 42). It is difficult to draw strong conclusions regarding the superiority of LVD to PVE because the studies included a small sample size of patients, lacked some outcomes, and presented essentially short-term follow-up. We should wait for more accurate data that will allow us to establish causality from the first French multicenter RCT HYPER-LIV01 trial (NCT03841305) and the Maastricht group DRAGON-2 (NCT05428735) comparing LVD and PVE in colorectal liver metastases. One additional point to consider was that the majority of studies were from Western centers and the translation of results could not be performed only by further studies from Eastern centers.

In conclusion, LVD is as feasible and safe as PVE with encouraging results making some selected patients more suitable for surgery, even with a small FLR. However, this interventional radiological procedure probably helps resolve the volumetric problem, but its effect on tumor progression remains to be better investigated.

## Data availability statement

The raw data supporting the conclusions of this article will be made available by the authors, without undue reservation.

## Author contributions

MC: Conceptualization, Data curation, Methodology, Resources, Software, Writing – original draft, Writing – review & editing. AM: Conceptualization, Data curation, Formal analysis, Writing – original draft. AC: Formal analysis, Funding acquisition, Resources, Writing – original draft. MH: Conceptualization, Investigation, Methodology, Resources, Validation, Writing – original draft. AG: Investigation, Resources, Supervision, Validation, Visualization, Writing – original draft. BK: Formal analysis, Funding acquisition, Investigation, Methodology, Resources, Software, Writing – original draft. FP: Methodology, Project administration, Resources, Software, Supervision, Validation, Visualization, Writing – review & editing. BG: Methodology, Project administration, Resources, Software, Supervision, Validation, Visualization, Writing – review & editing. OS: Methodology, Project administration, Resources, Software, Supervision, Validation, Visualization, Writing – review & editing. HO: Funding acquisition, Investigation, Methodology, Project administration, Resources, Software, Supervision, Validation, Visualization, Writing – review & editing.
